# The Role of EkoSonic Endovascular System or EKOS® in Pulmonary Embolism

**DOI:** 10.7759/cureus.6380

**Published:** 2019-12-14

**Authors:** Kashmala Khan, Deanna Yamamura, Carlos Vargas, Thomas Alexander, Salim Surani

**Affiliations:** 1 Internal Medicine, Corpus Christi Medical Center, Corpus Christi, USA; 2 Cardiology, Corpus Christi Medical Center, Corpus Christi, USA; 3 Internal Medicine, Texas A&M Health Science Center, Temple, USA

**Keywords:** pulmonary embolism, acute massive pulmonary embolism, submassive pe, ekos, catheter directed thrombolysis, ultrasound catheter directed therapy, catheter directed therapy, right heart strain

## Abstract

Pulmonary embolism has become a cause of great concern to health care professionals. Despite strides in research and availability of sensitive diagnostic tests, the mortality and morbidity related to this entity continues to cause tremendous economic burden. Patients present with an array of symptoms ranging from mild dyspnea to hemodynamic instability and even death. Prompt recognition of symptoms along with early risk stratification can be lifesaving. Management focuses on achieving hemodynamic stability and reducing clot burden. Approved treatment modalities include anticoagulation, systemic or catheter directed thrombolytic therapy and surgical embolectomy. In this article we will review catheter-directed thrombolytic therapy, specifically the EKOS® or the EkoSonic endovascular system. EKOS® uses ultrasound-facilitated catheter-directed thrombolysis. The rationale behind this therapy is using shorter infusion times and lower dosage of the thrombolytic therapy, thereby reducing the complications associated with their use.

## Introduction and background

Pulmonary embolism (PE) can be defined as the blockage of either the pulmonary arteries or its branches with embolic material (either air, fat, amniotic fluid or a thrombus) that originates elsewhere in the body. A thrombus from the deep veins of the lower extremities that embolizes is the most common culprit. Venous thromboembolism (VTE) consists of both pulmonary embolism and deep vein thrombosis (DVT), with the former being the more dreaded and severe presentation of VTE [1,2]. VTE, specifically PE, is the third most common cause of cardiovascular deaths worldwide, following myocardial infarction and cerebrovascular diseases [3]. In the United Sates, the incidence of VTE is about 900,000 which includes both DVT and PE, with PE accounting for about 150,000-250,000 hospitalizations, and about 60,000-100,000 deaths annually [4]. With one of the highest mortality and morbidity rates, PE has become a burden on the United Sates healthcare system. Currently VTE has an annual cost of $7-10 billion [5].

**Figure 1:**
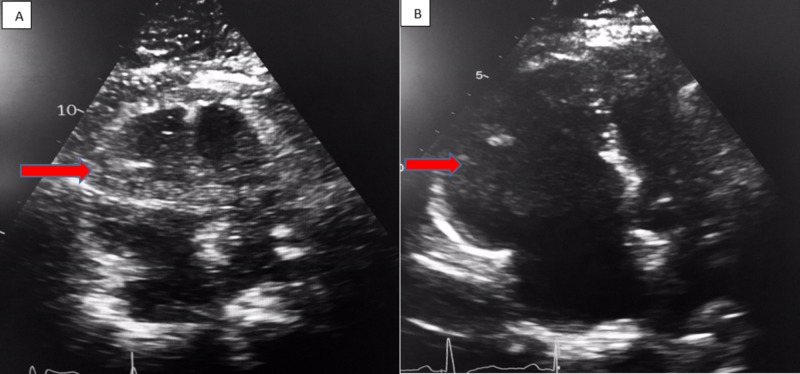
Apical four chamber view on transthoracic echocardiogram. (A) Shows increased RV:LV ratio (red arrow) in a patient with pulmonary embolism. (B) Shows increased RV:LV ratio of 1.8 (red arrow) in a patient with pulmonary embolism. RV: Right ventricular; LV: Left ventricular.

Presentation and clinical picture may vary, ranging from dyspnea, to sudden cardiac death which is seen in 25%-30% of patients with PE [2]. The treatment of pulmonary embolism depends on the pattern of presentation. It is imperative to establish hemodynamic status as it plays a critical role in the diagnosis, treatment and prognosis of patients with PE. Patients with hemodynamic instability are defined as having hypotension and are classified as having massive PE. The absence of hypotension but presence of right heart strain is known as submassive PE. One way to define right heart strain would be through echocardiography. On echocardiographic evaluation there would be an increase in right ventricular (RV) to left ventricular (LV) end diastolic diameter ratio of more than 1 on the apical four chamber view on transthoracic echocardiography (TTE) (Figure 1).

There would be presence of RV end diastolic diameter greater than 30 mm. Right heart strain causing inferior vena cava collapse can also be seen. There is RV free wall and base hypokinesis with sparing of the apex [2]. Biomarkers that are frequently seen are troponins which indicate myocardial injury and brain natriuretic peptide (BNP) or N-terminal pro BNP that suggests myocardial dilation [4]. The presence of both right heart strain and positive biomarkers has been associated with increased mortality [6].

## Review

Treatment modalities for PE include anticoagulation, systemic thrombolytic therapy, catheter-directed therapy (CDT) and catheter or surgical embolectomy. Anticoagulation is indicated in all patients presenting with confirmed PE unless there is an absolute contraindication. Anticoagulants can help prevent further clot formation. Anticoagulation can be achieved with vitamin K antagonists, low molecular weight heparin (LMWH), unfractionated heparin (UFH) and direct oral anticoagulants (DOACs) [7]. Thrombolytic therapy is used as an adjunctive therapy to anticoagulation to help reduce clot burden, by aiding in dissolving the current thrombus. Systemic thrombolytic therapy is indicated in patients with massive PE and significant clot burden [8]. According to the American College of Chest Physicians, thrombolytic therapy in massive PE is a class 2b indication for treatment, whereas CDT is a class 2C indication [9,10]. The rationale behind using catheter directed in place of systemic thrombolytic therapy is shorter infusion times and therefore less risk of bleeding complications from the thrombolytic agents. CDT modalities include direct fragmentation of the thrombus via a pigtail catheter, thrombus aspiration or suction embolectomy, rheolytic thrombectomy (use high flow saline jet), thrombolytic infusion catheters and ultrasound facilitated CDT therapy. This article focuses on EKOS® which is a type of ultrasound CDT [11].

**Figure 2:**
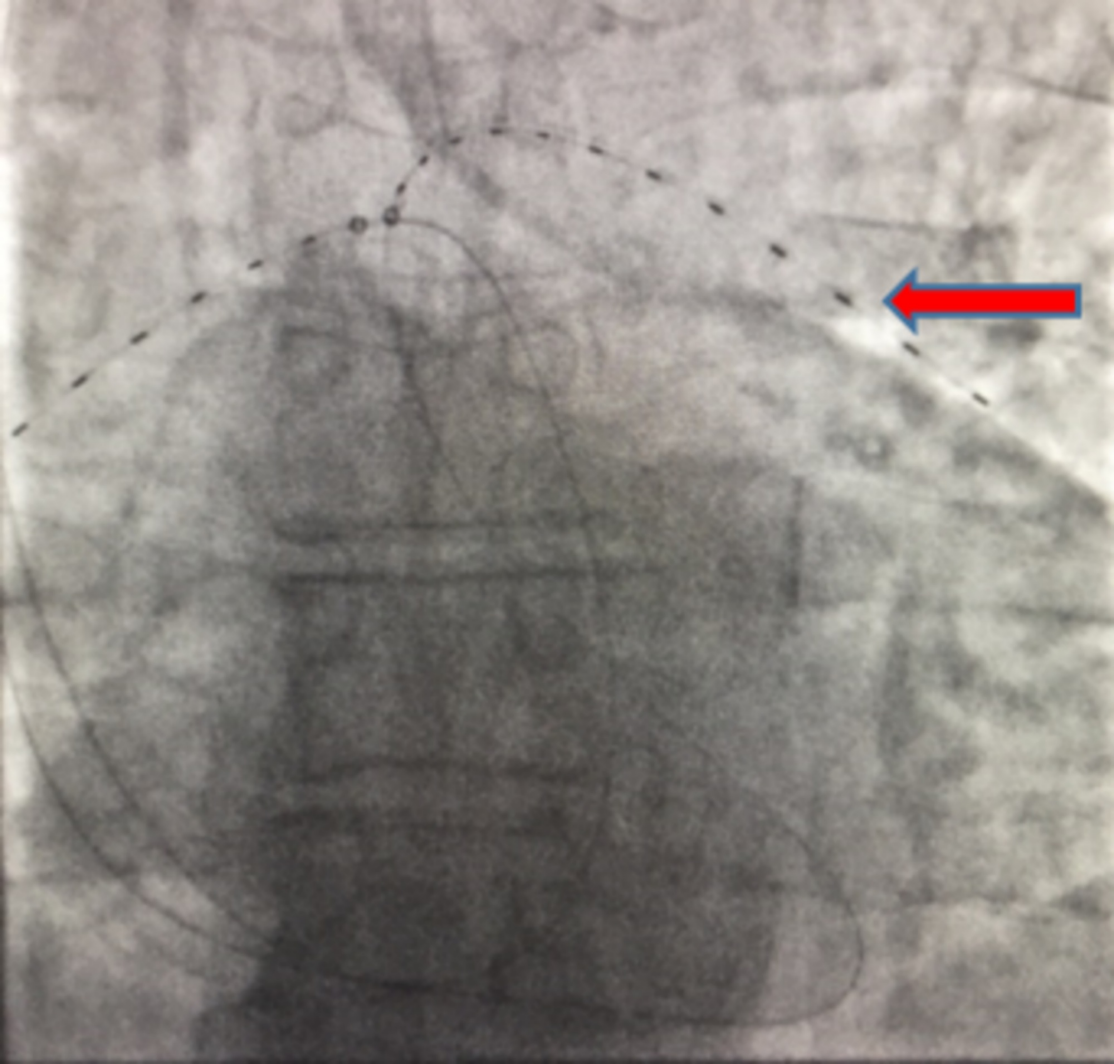
Bilateral EKOS catheters on fluoroscopy. Each marker is 1 cm (red arrow) and indicates an area of ultrasound.

The EKOS® or EkoSonic endovascular system was approved by the FDA in 2004 [12]. It is used for the treatment of PE, DVT and arterial occlusion. The EKOS system consists of an infusion catheter, an ultrasound core wire and a control unit (Figure 2).

This technology uses high frequency, low power ultrasound energy in combination with thrombolytic therapy to achieve clot dissolution. Catheter-directed thrombolysis requires thermal-mediated diffusion by which the thrombolytic agent is driven into the thrombus via a high concentration gradient. The ultrasound uses cavitation-induced microstreaming which loosens the fibrin strands within the clot and enhances mechanical breakdown of the thrombus. It also increases the surface area of the clot, as a result more active plasminogen receptor sites are in contact with the thrombolytic agent. In addition, the ultrasound enhances the transport of thrombolytic agent across the clot and makes it more permeable [12,13]. Major complication in using catheter-directed therapies is increased risk of bleeding from the thrombolytic agent itself. Other complications include hematoma at the site of access, injury to the pulmonary artery, pulmonary hemorrhage and retroperitoneal hemorrhage [10].

There have been multiple studies over recent years that have investigated the efficacy, benefits and adverse effects of thrombolytic therapy used in combination with anticoagulation versus anticoagulation alone (Table 1).

**Table 1:** Baseline characteristics of trials comparing anticoagulation and thrombolytic therapy. * CDT: Catheter-directed thrombolysis; EKOS®: EkoSonic endovascular system; FH: Fractionated heparin (i.e., enoxaparin); LV: Left ventricle; MPE: Massive pulmonary embolism; N: No. of patients; PE: Pulmonary embolism; RV: Right ventricle; SMPE: Submassive pulmonary embolism; tPA: tissue plasminogen activator; UFH: Unfractionated heparin; USCDT: Ultrasound facilitated catheter-directed thrombolysis. ^†^ Treatment arms: Arm 1 (4 mg/lung/2 h). Arm 2 (4 mg/lung/4 h). Arm 3 (6 mg/lung/6 h). Arm 4 (12 mg/lung/6 h).

Trial	N	Methods used in trial (N)		Summary
MOPETT (2013)	121	Low dose tPA plus AC (61)	AC (FH, UFH, warfarin) (60)	In patients with submassive PE, low dose tPA plus anticoagulation was found to reduce the incidence of pulmonary hypertension, lower pulmonary artery pressures and decreased hospital stay. No significant difference was noted in the rate of individual outcomes of death and recurrent PE when assessed independently.
PEITHO (2014)	1,005	Tenecteplase 30-50 mg IV once + UFH (506)	Placebo + UFH (499)	The treatment group was found to have reduced hemodynamic decompensation and all-cause mortality at seven days in patients with submassive PE. Patients in the treatment group experienced increased rates of major extracranial bleeding and strokes.
PERFECT (2015)	101	CDT MPE (73)	CDT SMPE (28)	CDT in patients with submassive PE or massive PE showed improvement of mean pulmonary artery pressures, and right-sided heart strain based on echocardiography. There were no major procedure-related complications, major hemorrhages, or hemorrhagic strokes.

The MOPETT trial (2013) was a randomized trial conducted to compare safe dose (half dose) thrombolytic in addition to anticoagulation with anticoagulation alone in 121 patients with sub-segmental PE. In the thrombolytic group there was lower incidence of pulmonary hypertension, lower pulmonary artery pressures and decreased hospital stay [14]. The PEITHO trial (2014) was a multicenter, double blinded placebo controlled randomized trial that enrolled 1005 patients that were treated with either thrombolytic and heparin or placebo and heparin. This trial found that in the thrombolytic group, there was decreased death and decompensation, but there was also increased incidences of stroke (mostly hemorrhagic) [15]. The PERFECT trial (2015) was able to study 101 patients with either massive or sub-massive PE who underwent CDT. This trial was able to conclude that patients undergoing CDT had improvement in right heart strain on echocardiography, decrease in pulmonary artery pressures, and minimal procedure-related complications and major bleeding [16].

The role of thrombolytic agents and anticoagulation has been well established according to the aforementioned trials and studies. Other trials and studies conducted have now included comparisons of ultrasound-facilitated CDT, and EKOS in particular (Table 2).

**Table 2:** Baseline characteristics of trials demonstrating the use of ultrasound CDT, particularly EKOS®. * CDT: Catheter-directed thrombolysis; EKOS®: EkoSonic endovascular system; FH: Fractionated heparin (i.e., enoxaparin); LV: Left ventricle; MPE: Massive pulmonary embolism; N: No. of patients; PE: Pulmonary embolism; RV: Right ventricle; SMPE: Submassive pulmonary embolism; tPA: tissue plasminogen activator; UFH: Unfractionated heparin; USCDT: Ultrasound-facilitated catheter-directed thrombolysis. ^†^ Treatment arms: Arm 1 (4 mg/lung/2 h). Arm 2 (4 mg/lung/4 h). Arm 3 (6 mg/lung/6 h). Arm 4 (12 mg/lung/6 h).

Trial	N	Methods used in trial (N)		Summary
SEATTLE II (2015)	150	CDT MPE (31)	CDT SMPE (119)	EKOS® decreased RV dilation, reduced pulmonary hypertension, decreased anatomic thrombus burden, and minimized intracranial hemorrhage in patients with massive and submassive PE.
ULTIMA (2014)	59	USCDT plus UFH	UFH	CDT with heparin utilizing EKOS® in patients with submassive PE was shown to decrease the RV/LV ratio within 24 hours of treatment. Additionally, a significant recovery of right ventricular systolic function was observed in the catheter-directed group when compared to heparin only group.
OPTALYSE (2018)	101	Patients received treatment with 1 of 4 regimens.†		Utilizing USCDT with low dose tPA and a shorter delivery in patients with submassive PE was associated with improved right ventricular function and reduced clot burden in comparison to baseline. One major intracranial hemorrhage due to USCDT did occur.

The SEATTLE II trial (2015), a prospective single-arm study, enrolled 150 patients with massive and submassive PE. It successfully demonstrated significant improvement in RV strain with reduction in RV/LV ratio, decrease in post procedure pulmonary hypertension and angiographic obstruction [17]. The ULTIMA trial (2014) observed 59 patients who either received heparin therapy alone or a combination of heparin, EKOS® and thrombolytic therapy. There was improvement in right ventricular dysfunction in patients with acute PE who received the combination therapy with heparin, EKOS® and thrombolytic therapy [18]. The OPTALYSE (2018) trial was a randomized cohort study that focused on 101 patients with submassive PE. This trial exhibited the use of that low dose, low duration of thrombolytic therapy was just as effective with using EKOS® as other regimens (studied in SEATTLE II and ULTIMA) [19]. A meta-analysis (2018) investigated 1168 patients with high and intermediate risk PE who received either CDT or ultrasound-facilitated CDT. In high-risk patients, clinical success rates were 70.8% for patients receiving CDT and 83.1% for patients receiving ultrasound CDT. In intermediate risk patients, the clinical success was 95% and 97.5% for CDT and ultrasound CDT, respectively. They concluded that ultrasound CDT may be more effective in high-risk patients [20].

Some studies done in the past few years, however, have shown similar results when comparing ultrasound CDT with standard CDT [21-23]. A retrospective study done compared patients with submassive and massive PE receiving either CDT or ultrasound CDT. Estimated 90-day survival for CDT and ultrasound CDT were 92% and 93%, respectively. Major and minor bleeding complications were 11% for CDT and 13.9 % for ultrasound CDT. This study was able to reach the conclusion that there was no statistically significant difference between the clinical outcomes or complication rates between the two groups [21]. Another retrospective study done analyzed 98 cases of massive PE, intermediate high- and low-risk patients who were either treated with CDT or ultrasound CDT. This study was able to demonstrate that there were similar improvements in hemodynamics and length of ICU and hospital stays in both treatment groups. They also observed lesser bleeding complications and survival-to-discharge in patients receiving CDT [22]. One single center retrospective study done reviewed 60 patients between 2010 and 2016. There were 52 patients with submassive PE and eight patients with massive PE. Patients underwent treatment with either pigtail CDT or ultrasound CDT. This study observed that both treatment modalities were equally effective in reducing pulmonary arterial pressures and miller index scores. They noted that fluoroscopy times were longer in the ultrasound CDT group, but complications and 30-day mortality rates were similar in both groups [23]. Although CDT is being used more frequently for submassive PE, physicians are still hesitant to use this treatment modality for patients presenting with massive PE, since the latter has a more dramatic clinical presentation. Most studies published have been from centers with more experience with these procedures, the results of which may not apply to smaller centers [24].

## Conclusions

CDT, and EKOS® in particular is evolving and becoming more popular for the treatment for PE. This can be attributed to the ultrasound waves aiding in clot fragmentation and dissolution, therefore requiring smaller dosage and shorter duration of thrombolytic therapy. With lower doses of thrombolytic therapy the bleeding risks are reduced but not entirely eliminated. CDT has been shown to improve right heart strain by reducing RV/LV ratios and pulmonary artery systolic pressures with an excellent safety profile. EKOS® requires expertise and manpower that not every institution is equipped with. The cost of this treatment modality is also one of the reasons it is not favored over standard CDT in some instances. Long-term benefits of EKOS® have yet to be established. While there is evident short-term benefit and significant clinical improvement, there is lack of prospective studies that follow these patients long term after receiving treatment. It would be valuable to recognize the rate of developing long-term complications of PE following EKOS® and CDT versus systemic thrombolysis.
